# Population Pharmacokinetics of Artesunate and Dihydroartemisinin following Intra-Rectal Dosing of Artesunate in Malaria Patients

**DOI:** 10.1371/journal.pmed.0030444

**Published:** 2006-11-28

**Authors:** Julie A Simpson, Tsiri Agbenyega, Karen I Barnes, Gianni Di Perri, Peter Folb, Melba Gomes, Sanjeev Krishna, Srivicha Krudsood, Sornchai Looareesuwan, Sharif Mansor, Helen McIlleron, Raymond Miller, Malcolm Molyneux, James Mwenechanya, Visweswaran Navaratnam, Francois Nosten, Piero Olliaro, Lorrin Pang, Isabela Ribeiro, Madalitso Tembo, Michele van Vugt, Steve Ward, Kris Weerasuriya, Kyaw Win, Nicholas J White

**Affiliations:** 1 Centre for Molecular, Environmental, Genetic and Analytic Epidemiology, The University of Melbourne, Melbourne, Australia; 2 School of Medical Sciences, Kwame Nkrumah University of Science and Technology, Kumasi, Ghana; 3 Division of Clinical Pharmacology, University of Cape Town, Cape Town, South Africa; 4 Clinica di Malattie Infecttive, Ospedale Amedeo di Savoia, Torino, Italy; 5 South African Medical Research Council, Tygerberg, South Africa; 6 UNICEF/UNDP/World Bank/WHO Special Programme for Research and Training in Tropical Diseases, World Health Organization, Geneva, Switzerland; 7 Division of Cellular and Molecular Medicine, Centre for Infection, University of London, London, United Kingdom; 8 Faculty of Tropical Medicine, Mahidol University, Bangkok, Thailand; 9 Centre for Drug Research, Universiti Sains Malaysia, Penang, Malaysia; 10 Pfizer Global Research and Development, Ann Arbor, Michigan, United States of America; 11 Malawi-Liverpool-Wellcome Trust Clinical Research Programme and Malaria Project, College of Medicine, Blantyre, Malawi; 12 Shoklo Malaria Research Unit, Mae-Sot, Tak, Thailand; 13 Wellcome Trust Mahidol University Oxford Tropical Medicine Research Programme, Mahidol University, Bangkok, Thailand; 14 Centre for Tropical Medicine and Vaccinology, Churchill Hospital, University of Oxford, United Kingdom; 15 Department of Health, Maui County, Hawaii, United States of America; 16 Department of AIDS and Tropical Medicine, Amsterdam Medical Center, Amsterdam, Netherlands; 17 Liverpool School of Tropical Medicine, Liverpool, United Kingdom; 18 Regional Office for South-East Asia, World Health Organization, New Delhi, India; 19 State Peace and Development Council, Myanmar; University of Western Australia, Australia

## Abstract

**Background:**

Intra-rectal artesunate has been developed as a potentially life-saving treatment of severe malaria in rural village settings where administration of parenteral antimalarial drugs is not possible. We studied the population pharmacokinetics of intra-rectal artesunate and the relationship with parasitological responses in patients with moderately severe falciparum malaria.

**Methods and Findings:**

Adults and children in Africa and Southeast Asia with moderately severe malaria were recruited in two Phase II studies (12 adults from Southeast Asia and 11 children from Africa) with intensive sampling protocols, and three Phase III studies (44 children from Southeast Asia, and 86 children and 26 adults from Africa) with sparse sampling. All patients received 10 mg/kg artesunate as a single intra-rectal dose of suppositories. Venous blood samples were taken during a period of 24 h following dosing. Plasma artesunate and dihydroartemisinin (DHA, the main biologically active metabolite) concentrations were measured by high-performance liquid chromatography with electrochemical detection. The pharmacokinetic properties of DHA were determined using nonlinear mixed-effects modelling. Artesunate is rapidly hydrolysed in vivo to DHA, and this contributes the majority of antimalarial activity. For DHA, a one-compartment model assuming complete conversion from artesunate and first-order appearance and elimination kinetics gave the best fit to the data. The mean population estimate of apparent clearance (CL/F) was 2.64 (l/kg/h) with 66% inter-individual variability. The apparent volume of distribution (V/F) was 2.75 (l/kg) with 96% inter-individual variability. The estimated DHA population mean elimination half-life was 43 min. Gender was associated with increased mean CL/F by 1.14 (95% CI: 0.36–1.92) (l/kg/h) for a male compared with a female, and weight was positively associated with V/F. Larger V/Fs were observed for the patients requiring early rescue treatment compared with the remainder, independent of any confounders. No associations between the parasitological responses and the posterior individual estimates of V/F, CL/F, and AUC_0–6h_ were observed.

**Conclusions:**

The pharmacokinetic properties of DHA were affected only by gender and body weight. Patients with the lowest area under the DHA concentration curve did not have slower parasite clearance, suggesting that rectal artesunate is well absorbed in most patients with moderately severe malaria. However, a number of modelling assumptions were required due to the large intra- and inter-individual variability of the DHA concentrations.

## Abstract 2

A study of the population pharmacokinetics of intra-rectal artesunate in patients with moderately severe falciparum malaria found the pharmacokinetic properties of dihydroartemisinin were affected only by gender and body weight.

## Editors' Summary

**Background.:**

More than 40% of the world's population is at risk of malaria, a tropical parasitic disease that is transmitted between people by infected mosquitoes. Malaria parasites cause a 'flu-like illness that includes chills, fevers, headaches, and sometimes nausea and vomiting. If untreated, people with malaria can rapidly become anemic—the parasite destroys their red blood cells—or can develop complications that damage the brain and other organs. Severe malaria can be fatal and must be treated quickly. It has become a matter of great concern that the parasite has developed resistance to most of the drugs used to treat or prevent malaria. In the past few years, artemisinin derivatives have been shown to be an effective new form of treatment. Artemisinin derivatives are effective, rapid-acting antimalarial drugs—wormwood, the plant source of artemisinin, is an ancient Chinese cure for malaria. Artesunate, a water-soluble derivative of artemisinin, can be given as tablets or as injections. However, people with severe malaria often cannot take oral medicines, and in rural settings in the developing world, artesunate injections are usually impracticable. Consequently, rectal artesunate suppositories have been developed to provide first-line treatment of severe malaria in these settings. This simple dosing method can “buy” patients valuable time during which they can be moved to a hospital for further treatment.

**Why Was This Study Done?:**

When treating severe malaria, it is important that every patient absorbs the antimalarial drug rapidly and efficiently into their blood. If even a small proportion of patients malabsorb the drug, many people could die. How the body processes a drug is known as pharmacokinetics, and although some pharmacokinetic studies have investigated how the body processes artesunate given in rectal suppositories, relatively little is know about the population pharmacokinetics of artesunate given this way. That is, the patient characteristics that affect the processing of intra-rectal artesunate are not known, and it is unclear whether a small proportion of the population might fail to absorb the drug given via this route. In this study, the researchers have developed and tested a population pharmacokinetic model for artesunate given rectally to children and adults with moderately severe malaria.

**What Did the Researchers Do and Find?:**

The researchers took serial blood samples from nearly 200 patients with moderately severe malaria in Africa and Southeast Asia for the first 24 hours after they received a rectal artesunate suppository. They measured the levels of artesunate and dihydroartemisinin (DHA; the body rapidly converts artesunate to DHA, which kills the malaria parasites) in these samples and used these data to build a pharmacokinetic model for how the body processes. Averaged out across the patients, they calculated, for example, that half of the drug present absorbed was eliminated within 43 minutes. To find out whether any patient characteristics affected the pharmacokinetics of intra-rectal artesunate, the researchers used their model to estimate the clearance of DHA from the body and the ability of DHA to spread through the body (so-called apparent volume of distribution) for the study patients. This analysis showed that only gender and weight affected DHA pharmacokinetics. Finally, the researchers showed that how well the parasite was cleared from the patients was not related to these pharmacokinetic parameters, although the need for earlier rescue treatment was associated with a larger volume of distribution for DHA. Importantly, the parasitological response was not affected by the estimated cumulative amount of DHA absorbed into the blood during the first six hours after treatment.

**What Do These Findings Mean?:**

The data presented in this study indicate that individual patients processed artesunate very differently in terms of how they absorbed the drug and how it spread around the body. Even so, the maximal effects of artesunate on the malaria parasite were achieved rapidly in nearly all the patients. This and other pharmacokinetic findings must be interpreted with caution, warn the researchers, because their model included many assumptions to allow, for example, for the variability of DHA concentrations both within individual patients and between patients. Nevertheless, the findings provide important clues about which patient characteristics might cause early treatment failure, and indicate that artesunate is sufficiently well absorbed via the rectal route in most patients to make artesunate suppositories a promising first-line treatment for moderately severe malaria.

**Additional Information.:**

Please access these Web sites via the online version of this summary at http://dx.doi.org/10.1371/journal.pmed.0030444.

• World Health Organization links to general information on malaria plus specific information on rectal artesunate

• MedlinePlus encyclopedia entry on malaria

• US Centers for Disease Control and Prevention information on malaria for patients and professionals

• Wikipedia pages on malaria and artemisinin (note that Wikipedia is a free online encyclopedia that anyone can edit)

## Introduction

Artemisinin and its derivatives are the most important class of antimalarial drug. Together with Cinchona alkaloids, they are the only drugs used to treat severe malaria when oral treatment is precluded, and recent evidence shows that parenteral artesunate (ARS) is the treatment of choice in adults hospitalised with severe malaria [1]. Although the parenteral route is preferred whenever facilities are available to diagnose and treat moderate or severe malaria, these facilities are usually lacking in the rural tropical villages where most cases arise. In these circumstances, the intra-rectal route for giving antimalarials has been suggested as an alternative to parenteral treatments [2–6]. Based on these observations, a development programme was implemented by World Health Organization–Special Programme for Research and Training in Tropical Diseases (WHO–TDR) in 1996 to define the pharmacokinetics, safety, and efficacy of intra-rectal ARS and to register an appropriate formulation for initial malaria treatment in situations in which oral treatment could not be taken yet facilities for parenteral treatment were unavailable. The reasons for supporting this strategy were: (1) facilities for parenteral treatment are lacking in most rural tropical settings where malaria first presents; (2) it may take many hours or even days for sick patients to reach facilities where malaria can be managed by adequate diagnostic testing and effective parenteral treatments; (3) ARS is the safest and most rapidly effective antimalarial available [7,8]; and (4) there was already clinical experience of the efficacy and safety of intra-rectal artemisinin suppositories.

The efficacy and safety of intra-rectal ARS has been studied in patients with malaria in Africa and Southeast Asia. Several conventional pharmacokinetic analyses and some population pharmacokinetic studies have been performed [9–12]. This paper aims to present a comprehensive population pharmacokinetic analysis of the Phase II and III studies performed both in children and adults with moderately severe malaria. Additionally, the relationship between pharmacokinetic parameters and important clinical and parasitological variables is assessed.

## Methods

Pharmacokinetic data were collected prospectively from two Phase II and three Phase III studies. One of these has been published previously after conventional pharmacokinetic analysis [10]. Each study was approved by the relevant local ethics committee, and all were approved by the Secretariat Committee on Research Involving Human Subjects (SCRIHS) of the World Health Organization, now named WHO Ethical Review Committee (ERC). Written informed consent was obtained from all patients or guardians/relatives. Details of study sites, patient age groups, and blood sampling schedules for drug assays are given in Table 1.

**Table 1:**
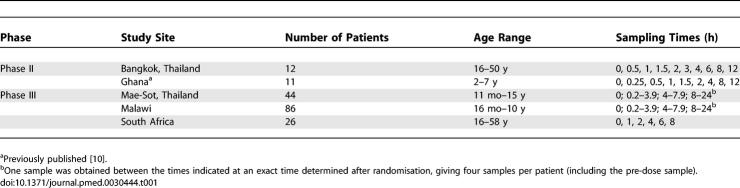
Study Sites and Sampling Times

### Patients

#### Inclusion criteria.

Patients in Ghana, Malawi, South Africa, and Bangkok had moderately severe falciparum malaria [13] (overall asexual parasite density range 0.1%–27%), were unable to tolerate oral medication, but did not show clinical signs or laboratory features of severe malaria (defined below). Patients from Mae-Sot, Thailand, were enrolled only if they had hyperparasitaemia defined as parasitaemia ≥4% [14]. Patients studied in Bangkok were enrolled only if parasitaemia exceeded 100,000/μl.

#### Exclusion criteria.

Patients with severe malaria (cerebral malaria, hypoglycaemia, hyperlactataemia, severe anaemia), recurrent convulsions, acute diarrhoea, diseases of the rectum, rectal surgery, known treatment with an effective antimalarial within 24 h of presentation, pregnant or breast feeding, with >20% parasitaemia, or with known hypersensitivity to the treatment drugs were excluded from this study. For exclusion purposes cerebral malaria was classified differently in the various countries (Malawi, Ghana, South Africa: Blantyre coma score <3; Mae-Sot: Blantyre coma score <5). The cut-off for severe anaemia was a haematocrit of 15% in South Africa and Mae-Sot; and 18% in Malawi, Bangkok, and Ghana. Patients with hyperlactataemia (defined as venous plasma lactate levels ≥5 mmol/l) were excluded only in the case of South Africa and Ghana. As lactate levels were not available at the time of enrolment in Malawi and the Thai studies, patients were not excluded on that basis.

#### Dosing.

All patients received the nearest approximation to 10 mg/kg intra-rectal ARS as a single dose (the range of actual doses by study site is given in Table 2). The dose formulations were suppositories of 50 mg and 200 mg (Mepha Pharmaceuticals, Aesch-Basle, Switzerland).

**Table 2:**
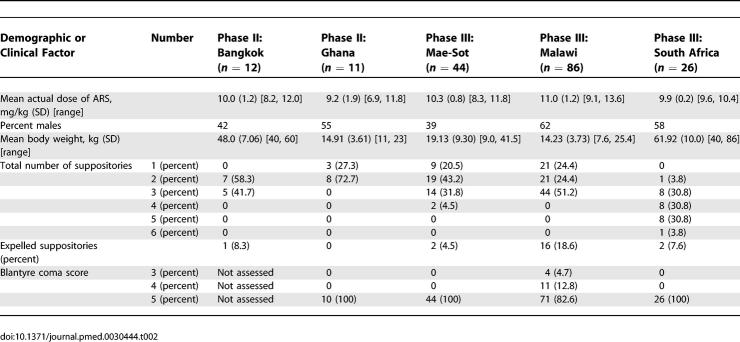
Demographic and Clinical Data of All Patients

There being no data on the reliability of drug absorption in this group of patients, all studies were open label, with rescue criteria specified for the ARS group on the grounds of clinical deterioration or unsatisfactory parasitological response in the Phase II trials in Bangkok and Ghana. In the trials in Malawi, South Africa, and Mae-Sot, patients treated with rectal ARS were to be rescued if their parasitaemia did not decline to below 60% of baseline parasitaemia at 12 h unless their condition was improving clinically. In Malawi and South Africa the patient was also treated with quinine if the patient subsequently developed any features of the exclusion criteria in the period of 24 h of observation following rectal treatment. Rescue criteria were specified for the comparator drug in the South African and Thai trials. Follow-up oral treatments were chloroquine (except in the case of chloroquine intolerance) in Ghana, sulphadoxine-pyrimethamine in Malawi and South Africa, oral ARS in Mae-Sot, and mefloquine in Bangkok.

### Collection and Analysis of Samples

In the Phase II studies, blood samples were taken at fixed intervals eight times (15 min, 30 min, 60 min, 90 min, 2 h, 4 h, 8 h, and 12 h) post treatment in Ghanaian children and nine times (30 min, 60 min, 90 min, 2 h, 3 h, 4 h, 6 h, 8 h, and 12 h) in Thai adults after administration of rectal ARS. In the Phase III South Africa study, timed sampling was taken at 1 h, 2 h, 4 h, 6 h, and 8 h post treatment. In parallel studies in Malawi and Mae-Sot, randomised sparse sampling was employed. Three plasma samples were taken for each individual recruited into the trial, the timing of each sample determined by randomisation within three blocks (0.2–3.9 h, 4–7.9 h, and 8–24 h) during the first 24-h period post treatment. Because drug concentrations were rarely measurable after 12 h, the protocol was amended during the study to sample between 0.25–1.9 h, 2–5.9 h, and 6–12 h.

Sample volumes obtained at each time point (Table 1) varied between 1 ml and 10 ml, depending upon the age of the patient. Whole blood was collected into appropriately labelled, heparinised (30IU) tubes, and plasma was separated within 30 min of collection by centrifugation at 1,000 × g for 15 min. Samples were stored in plastic cryotubes (Nalgene, Rochester, New York, United States), transported on dry ice, and stored below −70 °C. The longest interval between sample collection and analysis was 6 mo.

Concentrations of ARS and dihydroartemisinin (DHA) in plasma were determined by a specific and sensitive high-performance liquid chromatographic method with ElectroChemical Detector (Model BAS 200A, West Lafayette, Indiana, United States) operating in the reductive mode, as described previously [15]. All samples were assayed at the same laboratory. The mean recovery of ARS and DHA over a concentration range of 50–200 ng/ml was 75.5% and 93.5%, respectively. The within-day coefficients of variation were 4.2–7.4% for ARS and 2.6–4.9% for DHA. The day-to-day coefficients of variation were 1.6–9.6% and 0.5–8.3%, respectively. The minimum detectable concentration for both ARS and DHA in plasma was 8 ng/ml. The upper limit of the assay was 3,200 ng/ml with a limit of quantification of 50 ng/ml for all study sites except Mae-Sot, where the limit of quantification was 20 ng/ml. All samples were analysed according to principles and standards of Good Laboratory Practice.

### Population Pharmacokinetic Modelling

One-compartment and two-compartment models were fitted to the ARS and DHA concentrations. It was assumed that ARS was converted completely to DHA (i.e., there was no other significant route of ARS elimination) for determination of the dose in the pharmacokinetic analyses of the DHA concentrations. The exact dose (mg/kg) administered was used in the modelling. Inter-individual variability in the pharmacokinetic parameters was modelled with log-normal error models.

For example:

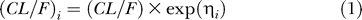
where *CL/F_i_* is the pharmacokinetic parameter for the individual “i” and *CL/F* is the population mean. The *η_i_^CL/F^* is the random effect with zero mean and variance σ^2^_CL/F_, which represents the inter-individual variability for the parameter.

The magnitude of the inter-individual variability was expressed as a coefficient of variation (%CV; approximated by the square root of the variance estimate).

Various models for the residual intra-individual error (ɛ_ij_) were compared. Drug concentrations that were below the limit of quantification (LOQ) or above the assay range (i.e., >3,200 ng/ml) were included in the analyses because a large number of these levels are present and exclusion would reduce the power of the statistical modelling. A covariate representing the type of drug level (i.e., normal level versus below-LOQ level versus above-range level) was explored to investigate the differences in the magnitude of the residual intra-individual error.

The NLME procedure [16] of the S-PLUS data programme (S-PLUS 6.2 for Windows, Insightful, Seattle, Washington, United States) was used to calculate estimates of the population pharmacokinetic parameters, and the variances of the inter-patient and intra-patient (ɛ) error. Convergence was achieved when the objective value did not differ by more than a pre-specified difference (0.0001) and the programme returned the final estimates of the population pharmacokinetic parameters. The objective function (minus twice the log-likelihood of the data) was used to determine the model that best fitted the data. A significant drop in the objective function (using the chi-squared distribution with degrees of freedom equal to number of parameters that are set equal to a fixed value in the restricted model) from the general model to the restricted model was used to determine the final pharmacokinetic model. The goodness of fit of each model was also determined by the precision of the parameter estimates and examination of the scatter plot of residuals versus predicted concentrations. The individual pharmacokinetic parameters were calculated using the posterior estimates.

### Covariates

The covariates recorded in all studies and investigated were age, body weight (kg), gender, geographic group (Southeast Asia versus Africa), number of suppositories, baseline parasitaemia (/μl), baseline venous plasma lactate (mmol/l), baseline plasma glucose (mmol/l), and baseline packed cell volume (PCV). Baseline lactate and glucose levels were not measured at the Mae-Sot study site. Therefore, the patients from Mae-Sot were excluded from the covariate analysis of lactate and glucose. All covariates selected were deemed important a priori by the study investigators.

In the population pharmacokinetic modelling, covariates that were continuous variables (except weight) were centred around their median values so that the population estimates would represent those of an average patient. Body weight was included in the model using allometric scaling, i.e., (weight/70) for V/F and (weight/70)^0.75^ for CL/F. A statistically significant improvement in the objective function, improvement in the precision of the parameter estimate (standard error), and reduction in inter-individual and intra-individual variability were used to determine the importance of the covariates as predictors.

### Pharmacodynamics

To investigate the relationship between pharmacokinetic parameters (posterior individual estimates of CL/F, V/F, and area under the plasma concentration time curve (AUC) in the first 6 h [AUC_0–6h_]) and pharmacodynamics of the drug, the following prospectively defined pharmacodynamic measures were explored: (a) Treatment failure (death or malaria positive smear from day 7 to 28 or undetermined response [no smear or drop out]) versus success (survival and negative smear from day 7 to 28). The rectal treatment was not a curative treatment, so recrudescence depended also on subsequent treatment. (b) Whether the patient received rescue treatment (absconded or death or rescued versus not rescued). (c) Failure of baseline parasitaemia to fall 40% by 12 h (PC60). (d) Time for baseline parasitaemia to fall by 50% (PCT_50_) and 90% (PCT_90_). These parasite clearance times were determined from parasite counts recorded 6-hourly for all study sites except Ghana, where the parasite count of each child was determined every 4 h.

Multiple linear or logistic regressions, where appropriate, were performed to model the relationship between the pharmacodynamic measures (PCT_50_, PCT_90,_ treatment outcome, early rescue treatment, PC60), and the independent variables: posterior individual estimates of CL/F, V/F, and AUC_0–6h_, parasitaemia at admission (log transformed), baseline packed-cell volume, age, and gender.

## Results

Demographic, clinical, parasitological, and laboratory data for all patients by study site are presented in Tables 2 and 3. All patients fulfilled the criteria for moderately severe malaria with parasitaemias >0.1%, moderately elevated plasma lactate levels (except in Bangkok and Malawi, where three and seven patients, respectively, had lactate concentrations >5 mmol/l), and moderate anaemia. The total number of suppositories given to each patient was usually between one and three (89%), although up to six suppositories were given to South African adults. Few (<10%) suppositories were expelled, the exception being Malawi, where 19% of children expelled suppositories.

**Table 3:**
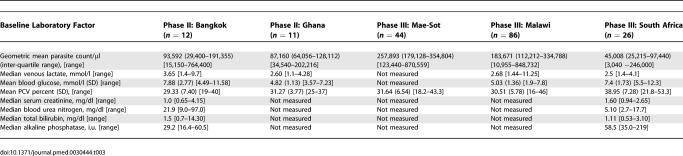
Baseline Laboratory Data of All Patients

### Safety and Clinical Efficacy

One patient from Mae-Sot died. The most likely cause of death was considered to be over-transfusion of 5% dextrose leading to iatrogenic water intoxication. Nineteen patients required rescue treatment (Table 4).

**Table 4:**
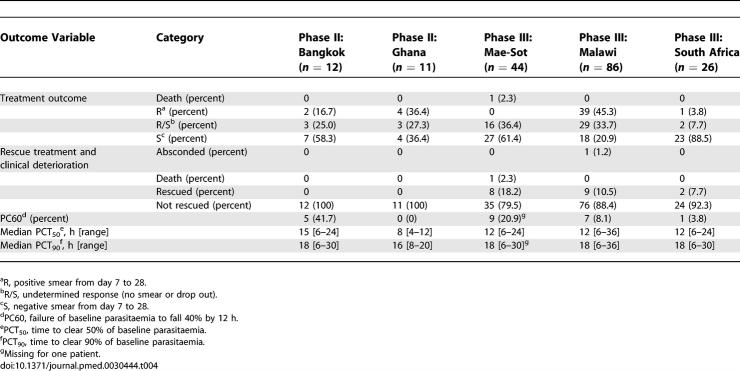
Efficacy Data of All Patients

The integrated adverse-event count yielded 66 adverse events from the 179 patients. The leading adverse events reported were gastrointestinal (mostly nausea, vomiting, and abdominal pain). For five patients, central nervous system or neurological adverse events were observed. These were convulsions, dizziness, impairment of consciousness, abnormal reflexes, and vertigo. However, none of the reported events led to treatment discontinuation or were classified as causally related to rectal ARS therapy.

### Artesunate Pharmacokinetics

A total of 307 levels from 136 patients were available for the statistical modelling (one outlier with an ARS concentration of 9,224 ng/ml 13 min after drug administration was excluded because of the likelihood of sample contamination). Of these, 32 (10.4%) levels were below the limit of quantification and one (0.3%) level was above the range.

**Figure 1:**
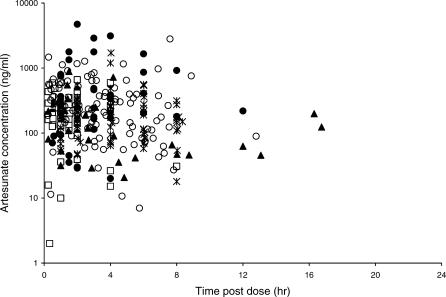
Observed Artesunate Concentrations (Semilogarithmic Scale; ng/ml) versus Time post Dose (h) Study site: filled circle, Bangkok; open square, Ghana; filled triangle, Mae-Sot; open circle, Malawi; asterisk, South Africa.

Figure 1 shows a scatter plot of the observed ARS concentrations versus time, by study site. There are no major differences in the distribution of data across the five different study sites. A clear pharmacokinetic profile is difficult to discern, precluding formal pharmacokinetic modelling. The median (range) of the observed individual peak concentrations was 269 ng/ml (11–4,720 ng/ml).

### DHA Pharmacokinetics

**Figure 2:**
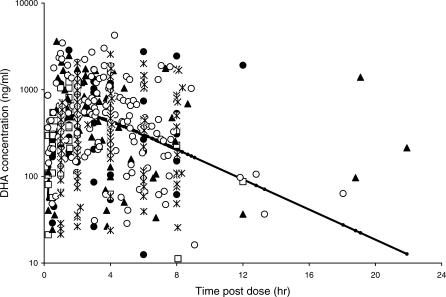
Observed and Population Predicted Concentrations (Solid Black Line) of DHA (Semilogarithmic Scale; ng/ml) versus Time post Dose (h) Study site: filled circle, Bangkok; open square, Ghana; filled triangle, Mae-Sot; open circle, Malawi; asterisk, South Africa. The population predicted concentrations are calculated for a dose of 10 mg/kg.

A total of 424 levels were available for statistical modelling from 164 patients (three outliers were excluded with levels >6,000 ng/ml). Of these, 33 (7.8%) levels were below the limit of detection and three (0.7%) were above the range. Figure 2 shows a scatter plot of the observed DHA concentrations versus time, by study site. A one-compartment model with first-order appearance and elimination kinetics including lag time best fits the data (appearance rate was fixed at 0.2/h and lag time at 0.14 h).

**Figure 3:**
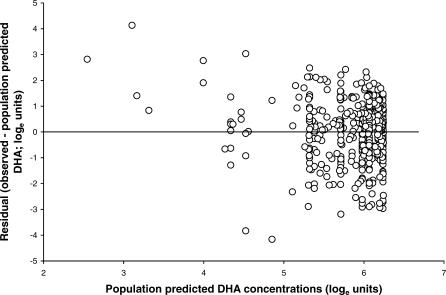
Residual (log_e_ Units) versus Population Predicted Concentrations of DHA (log_e_ Units) Residual = observed − population predicted concentration of DHA.

The data did not support a full variance–covariance matrix for the random effects of CL/F and V/F. The mean population estimate of CL/F was 2.64 (l/kg/h) with 66% inter-patient variability, and V/F was 2.75 (l/kg) with 96% inter-patient variability. These estimates correspond to an elimination half-life of 43 min. The residual error model that gave the minimum value for the objective function was a lognormal residual error model for all the data. Including a covariate for “study site” or “levels outside the limits of the assay” in the residual error modelling did not improve the objective function. Figure 2 shows the observed and population-predicted concentrations versus time. The residual plot indicated no bias in estimation (see Figure 3), and the patient-specific profiles were characterised adequately (unpublished data).

### Relationship between DHA Pharmacokinetic Variables and Covariates

A total of 164 of the 179 patients had posterior individual estimates of CL/F and V/F derived from the population pharmacokinetic modelling of DHA.

### Age, Body Weight, Gender, and Geographic Group (Southeast Asia versus Africa)

When age was treated as a dichotomous variable, children (age ≤15 y) versus adults (age >15 y), there was no significant difference between adults and children for the distribution of the patient-specific deviations from the population estimate of CL/F (η_CL/F_), whereas the patient-specific deviations from the population estimate of V/F (η_V/F_) were higher for adults compared with children (*p* < 0.001). No correlation was observed between η_CL/F_ and body weight, whereas there was a positive correlation between η_V/F_ and body weight (*r* = 0.42). There was no significant difference between males and females for the distribution of η_V/F_, whereas significantly higher values of η_CL/F_ were observed for males compared with females (*p* = 0.003). There were no significant differences between patients from Southeast Asia and Africa, for both the distributions of η_CL/F_ and η_V/F_.

### Baseline Parasitaemia, PCV, Lactate and Glucose, and Number of Suppositories

No correlations were observed between η_CL/F_ and η_V/F_ and baseline parasitaemia, lactate and glucose, and baseline PCV and η_CL/F_. There was a positive correlation between baseline PCV and η_V/F_ (*r* = 0.22, *p* = 0.005). There were no significant differences between both the distributions of η_CL/F_ and η_V/F_ for the number of suppositories given (either one, two, or three or more).

Table 5 presents the final population parameter estimates and the inter-individual and intra-individual variability. Including the covariates “gender” and “body weight” reduced the inter-individual variability of CL/F from 66% to 62% and V/F from 96% to 75%, respectively. The addition of baseline PCV and age did not significantly improve the objective function.

**Table 5:**
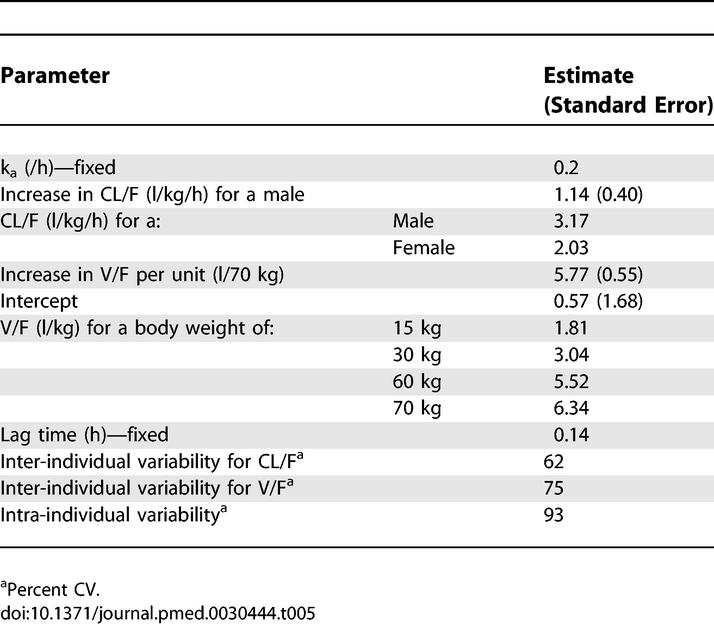
Population Pharmacokinetic Parameters for DHA

### Relationship between DHA Pharmacokinetic Parameters and Pharmacodynamic Variables

The pharmacodynamic variables defined prospectively as important were treatment outcome, early rescue treatment, failure of baseline parasitaemia to fall 40% by 12 h (PC60), and time to clear 50% (PCT_50_) and 90% (PCT_90_) of baseline parasitaemia.

The DHA pharmacokinetic parameters (available for 164 of the 179 study participants) investigated were posterior individual estimates of CL/F, V/F, and AUC_0–6h_.

### Treatment Outcome

For the statistical modelling, treatment outcome was a dichotomous variable with death, positive smear from day 7 to day 28, or undetermined response grouped together (termed “treatment failure”, *n* = 91) versus “treatment success” (negative smear from day 7 to day 28; *n* = 73). This definition of treatment outcome does not include the “early rescues”, which are considered under the “Early rescue treatment” section below.

No significant difference in the distribution of the posterior individual estimates of CL/F and AUC_0–6h_ was observed between the two groups of patients, treatment failure, and treatment success. Patients who had failed their treatment (*n* = 91) had significantly lower values of V/F compared with those with treatment success (*n* = 73) (geometric mean [95% CI]: 2.46 [2.25, 2.66] versus 3.16 [2.80, 3.60] l/kg; *p* = 0.001), but after multiple logistic regression analysis, with adjustment for baseline parasitaemia, baseline PCV, age, and gender (i.e., pre-defined confounders), no significant association between V/F and “treatment outcome” was observed (*p* = 0.12). The observed univariate association between “treatment outcome” and V/F was confounded by age.

### Early Rescue Treatment

**Figure 4:**
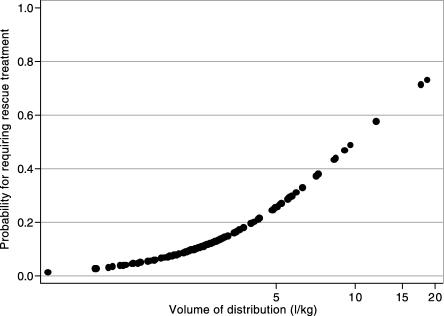
Relationship of Estimated Probability of Requiring Rescue Treatment and V/F of DHA (l/kg) Derived from a multiple logistic regression model with adjustment for the confounders: baseline parasitaemia, baseline PCV, age, and gender.

For the statistical modelling, rescue treatment was a dichotomous variable with absconded, death, or rescued by day 7 grouped together (*n* = 16) versus not rescued (*n* = 148). No significant association between rescue treatment and both CL/F and AUC_0–6h_ was observed. Those rescued had slightly higher values of V/F compared with those patients not requiring rescue treatment (geometric mean [95% CI]: 3.42 [2.58, 4.53] versus 2.69 [2.49, 2.90] l/kg, *p* = 0.056). From the multiple logistic regression analysis with adjustment for confounders, a significant association between V/F and “rescue treatment” was observed (*p* = 0.01). As the V/F increased, so did the risk of requiring rescue treatment (see Figure 4). Whether this reflected a truly larger apparent volume of distribution or a reduced fraction of drug absorbed (F) cannot be distinguished.

### Failure of Baseline Parasitaemia to Fall 40% by 12 h

There were 20 patients whose parasitaemia failed to drop by 40% of baseline values by 12 h. No significant association between failure and both CL/F and AUC_0–6h_ was observed, but patients who failed to drop parasitaemia by 40% by 12 h had higher V/F values compared with the remainder (geometric mean [95% CI]: 3.36 [2.17, 3.63] versus 2.67 [2.44, 2.80] l/kg; *p* = 0.045). Multiple logistic regression analysis with adjustment for confounders showed no significant association between V/F and “failure to drop to 60% parasitaemia at 12 h” (*p* = 0.09).

### Time to Clear 50% (PCT_50_) and 90% (PCT_90_) of Baseline Parasitaemia

**Figure 5:**
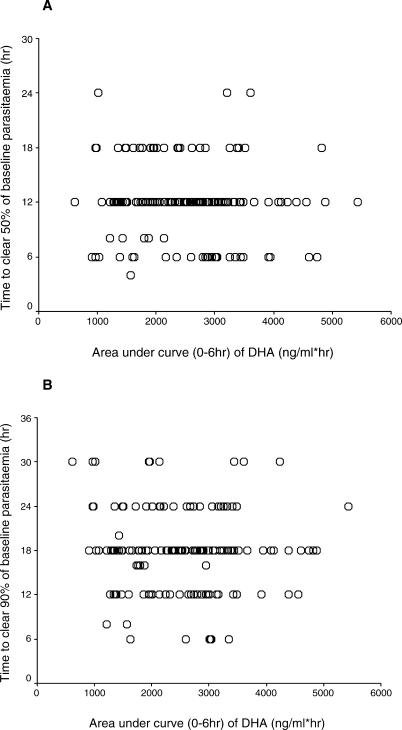
Observed PCT_50_ (A) and PCT_90_ (B) versus Posterior Estimate of DHA AUC_0–6h_ (ng/ml × h) For 162 patients (two patients excluded due to baseline parasitaemia <10,000/μl).

Two adult patients from South Africa with baseline parasitaemia <10,000/μl were excluded from the analyses of the efficacy variables, PCT_50_ and PCT_90_. No significant correlations between both PCT_50_ and PCT_90_ and the pharmacokinetic parameters CL/F, V/F, and AUC_0–6h_ were observed (Spearman rank correlation coefficients ranged from −0.07 to 0.17). These results were unchanged when adjusting for baseline parasitaemia, PCV, gender, and age. Figures 5A and 5B illustrate the lack of association between PCT_50_, PCT_90_, and AUC_0–6h_, respectively.

As ARS is rapidly hydrolysed in vivo to DHA and both have approximately equal antimalarial activity, the molar sums of ARS plus DHA plasma concentrations were also modelled. A total of 442 ARS-plus-DHA levels from 168 patients were available. The associations between the posterior individual PK parameters of ARS plus DHA (i.e., CL/F, V/F, and AUC_0–6_) and the efficacy variables (defined above) were the same as observed for the posterior individual PK parameters of DHA alone (unpublished data).

## Discussion

Malaria can evolve rapidly from uncomplicated to severe disease and death if not properly treated. Patients who cannot take oral treatment reliably are in serious danger and must be treated without delay. Rectal ARS provides the most rapidly effective available antimalarial in a simple and easily administered formulation. Earlier conventional pharmacokinetic studies compared 10-mg/kg doses and 20-mg/kg doses in both adults and children, and showed no significant pharmacodynamic differences between the two treatment groups and gave no safety concerns [10]. But in treating a rapidly life-threatening infection, defining the speed and reliability of absorption is critical. If even a small proportion of patients malabsorb the drug, this would be a major concern.

This pharmacokinetic analysis aimed to develop and test a population pharmacokinetic model of intra-rectal ARS, using data obtained from studies that were coordinated in design and analysis. Patient samples were handled in a standardised fashion and drug assays performed in one laboratory, to minimise assay variability. In addition to demographic variables and clinical and parasite response, it was anticipated that the number of suppositories and severity of disease might influence bioavailability. Bioavailability in patients failing treatment—judged either through parasitological effect or clinical response—was considered critical; therefore, a sample for pharmacokinetic analysis was obtained just prior to rescue, for such patients.

As other pharmacokinetic studies of ARS have observed [11], compartmental modelling for ARS data requires frequent sampling [17,18] because ARS is rapidly and extensively metabolised to the active metabolite, DHA [19,20]. Our data consisted of a mixture of rich and sparse sampling with considerable inter-patient variability in the ARS concentrations, which has been observed elsewhere [9,10,21], and as a result it was not possible to fit a pharmacokinetic model to the data. ARS concentrations were still observed 6 h post dosage in some patients in this dataset, suggesting that absorption may have become rate-limiting for ARS clearance. This has also been observed for parenteral ARS [18,22], and differs from oral administration, which produces lower and shorter-lived ARS levels.

The model that best fitted the DHA data was a one-compartment model with first-order appearance and elimination kinetics including lag time. Due to the sparseness and erratic nature of the data, both the appearance rate constant and lag time were fixed so that a satisfactory model fit could be obtained. Other studies have also used one-compartment models for DHA pharmacokinetics [10,23]. However, the choice of this model may reflect relatively lower sampling frequency, as more frequent sampling identified two-compartment modelling as being more appropriate in analysing DHA after parenteral ARS [18]. The mean population estimate of CL/F was 2.64 (l/kg/h), and volume of distribution (V/F) was 2.75 (l/kg), giving an elimination half-life of 43 min for DHA. These estimates are similar to those reported previously [10,11,23]. The fixed value of the appearance rate constant was slower than the estimated elimination rate constant, suggesting that the ARS rectal capsules are absorption rate limited. Therefore, the appearance rate of DHA will include both the absorption and elimination of ARS. The population-predicted profile of DHA was similar to that observed by Karunajeewa et al [11], with the maximum concentration occurring at approximately 3 h.

We observed a high intra-individual variability (93%) of DHA, as well as considerable inter-individual variability for both CL/F (66%) and V/F (96%). The F is not known; therefore, an increase in estimated CL/F or V/F could result from either an increase in clearance or volume or a reduction in F. Additionally, since it was not possible to estimate the appearance rate constant and the inter-individual variability of the appearance rate constant, changes in V/F may be due to changes in the appearance rate constant. This would have implications for treatment of patients with severe malaria, in whom speed of absorption is vital. Gender was observed to have a significant effect on CL/F, giving population estimates for CL/F of 3.17 (l/kg/h) and 2.03 (l/kg/h) for males and females, respectively. Body weight was observed to have a significant effect on V/F: the population estimates of V/F were 1.81 (l/kg) for a body weight of 15 kg, 3.04 l/kg for 30 kg, 5.52 l/kg for 60 kg, and 6.34 l/kg for 70 kg. Patients from different geographic areas were not observed to have different pharmacokinetic parameters.

Higher DHA V/Fs were observed for patients requiring “rescue treatment” compared with those patients not requiring rescue treatment, independent of the confounders, baseline parasitaemia, baseline PCV, age, and gender. Although this may reflect either reduced absorption (i.e., smaller F) or a truly larger apparent volume of distribution, further investigation to relate initial plasma concentrations of DHA and ARS + DHA (using AUC in the first 6 h) to parasite reduction failed to show significant relationships. The principal, directly measurable, pharmacodynamic effect of these compounds is to reduce parasitaemia rapidly by accelerating ring form clearance. Thus, inadequate early absorption would be expected to lead to a relationship between the AUC for ARS and DHA together and rate of parasitaemia reduction. This was not seen; patients with the lowest AUCs did not have discernibly slower parasite clearance. This suggests that maximal effects are achieved rapidly in nearly all patients, and that rectal ARS is a promising treatment for moderately severe malaria. However, these pharmacokinetic findings should be interpreted with caution: (i) the appearance rate constant of DHA was fixed in the modelling, (ii) there was large inter- and intra-patient variability in both the ARS and DHA concentrations, (iii) it was not possible to derive the pharmacokinetics of ARS, and (iv) the DHA pharmacokinetic parameters were influenced by both the absorption and clearance of ARS.

## Abbreviations:

ARS: artesunate
AUC: area under the plasma concentration time curve
CL/F: apparent clearance
DHA: dihydroartemisinin
F: fraction of drug absorbed
PCV: packed cell volume
V/F: volume of distribution

## References

[pmed-0030444-b001] Dorndrop A, Nosten F, Stepniewska N, Day NP, White NJ (2005). Artesunate versus quinine for treatment of severe falciparum malaria: A randomised trial. Lancet.

[pmed-0030444-b002] Li GQ, Guo XB (1990). Clinical studies on artemisinin suppositories, artesunate and artemether. Clinical trials on Qinghaosu and its derivatives. Volume 1.

[pmed-0030444-b003] Arnold K, Tran TH, Nguyen TC, Nguyen HP, Pham P (1990). A randomised comparative study of artemisininine (qinghaosu) suppositories and oral quinine in acute falciparum malaria. Trans R Soc Trop Med Hyg.

[pmed-0030444-b004] Cao XT, Bethell DB, Pham TP, Ta TT, Tran TN (1997). Comparison of artemisinin suppositories, intramuscular artesunate and intravenous quinine for the treatment of severe childhood malaria. Trans R Soc Trop Med Hyg.

[pmed-0030444-b005] Hien TT, Tam DT, Cuc NT, Arnold K (1991). Comparative effectiveness of artemisinin suppositories and oral quinine in children with acute falciparum malaria. Trans R Soc Trop Med Hyg.

[pmed-0030444-b006] Hien TT, Arnold K, Vinh H, Cuong BM, Phu NH (1992). Comparison of artemisinin suppositories with intravenous artesunate and intravenous quinine in the treatment of cerebral malaria. Trans R Soc Trop Med Hyg.

[pmed-0030444-b007] Myint HY, Tipmanee P, Nosten F, Day NP, Pukrittayakamee S (2004). A systematic overview of published antimalarial drug trials. Trans R Soc Trop Med Hyg.

[pmed-0030444-b008] Price R, van Vugt M, Phaipun L, Luxemberger C, Simpson J (1999). Adverse effects in patients with acute falciparum malaria treated with artemisinin derivatives. Am J Trop Med Hyg.

[pmed-0030444-b009] Ashton M, Nguyen DS, Nguyen VH, Gordi T, Trinh NH (1998). Artemisinin kinetics and dynamics during oral and rectal treatment of uncomplicated malaria. Clin Pharmacol Ther.

[pmed-0030444-b010] Krishna S, Planche T, Agbenyega T, Woodrow C, Agranoff D (2001). Bioavailability and preliminary clinical efficacy of intrarectal artesunate in Ghanaian children with moderate malaria. Antimicrob Agents Chemother.

[pmed-0030444-b011] Karunajeewa HA, Ilett KF, Dufall K, Kemiki A, Bockarie M (2004). Disposition of artesunate and dihydroartemisinin after administration of artesunate suppositories in children from Papua New Guinea with uncomplicated malaria. Antimicrob Agents Chemother.

[pmed-0030444-b012] Navaratnam V, Mansor SM, Sit NW, Grace G, Qiqui L (2000). Pharmacokinetics of artemisinin-type compounds. Clin Pharmacokinet.

[pmed-0030444-b013] Newton CR, Krishna S (1998). Severe falciparum malaria in children: Current understanding of pathophysiology and supportive treatment. Pharmacol Ther.

[pmed-0030444-b014] Luxemburger C, Nosten F, Raimond SD, Chongsuphajaisiddhi T, White NJ (1995). Oral artesunate in the treatment of uncomplicated hyperparasitemic falciparum malaria. Am J Trop Med Hyg.

[pmed-0030444-b015] Navaratnam V, Mordi MN, Mansor SM (1997). Simultaneous determination of artesunic acid and dihydroartemisinin in blood plasma by high-performance liquid chromatography for application in clinical pharmacological studies. J Chromatogr B Biomed Sci Appl.

[pmed-0030444-b016] Lindstrom ML, Bates DM (1990). Nonlinear mixed effects models for repeated measures data. Biometrics.

[pmed-0030444-b017] Batty KT, Thu LT, Davis TM, Ilett KF, Mai TX (1998). A pharmacokinetic and pharmacodynamic study of intravenous vs. oral artesunate in uncomplicated malaria. Br J Clin Pharmacol.

[pmed-0030444-b018] Nealon C, Dzeing A, Muller-Romer U, Planche T, Sinou T (2002). Intramuscular bioavailability and clinical efficacy of artesunate in Gabonese children with severe malaria. Antimicrob Agents Chemother.

[pmed-0030444-b019] Li QG, Peggins JO, Fleckenstein LL, Masonic K, Heiffer MH (1998). The pharmacokinetics and bioavailability of dihydroartemisinin, arteether, artemether, sodium artesunate and artelinate in rats. J Pharm Pharmacol.

[pmed-0030444-b020] Titulaer HA, Zuidema J, Kager PA, Wetsteyn JC, Lugt CB (1990). The pharmacokinetics of artemisinin after oral, intramuscular and rectal administration to volunteers. J Pharm Pharmacol.

[pmed-0030444-b021] Halpaap B, Ndjave M, Paris M, Benakis A, Kremsner PG (1998). Plasma levels of artesunate and dihidroartemisinin in children with Plasmodium falciparum malaria in Gabon, after administration of 50 mg artesunate suppositories. Am J Trop Med Hyg.

[pmed-0030444-b022] Mithwani S, Aarons L, Kokwaro GO, Majid O, Muchohi S (2004). Population pharmacokinetics of artemether and dihydroartemisinin following single intramuscular dosing of artemether in African children with severe falciparum malaria. Br J Clin Pharmacol.

[pmed-0030444-b023] Bethell DB, Teja-Isavadharm P, Cao XT, Pham TT, Ta TT (1997). Pharmacokinetics of oral artesunate in children with moderately severe Plasmodium falciparum malaria. Trans R Soc Trop Med Hyg.

